# Plasma microRNA expression profiles in Chinese patients with rheumatoid arthritis

**DOI:** 10.18632/oncotarget.6449

**Published:** 2015-12-02

**Authors:** Wenhong Wang, Yingying Zhang, Bo Zhu, Tanghai Duan, Qiugui Xu, Rui Wang, Liwei Lu, Zhijun Jiao

**Affiliations:** ^1^ Zhenjiang Key Laboratory of Medical Immunology, Department of Laboratory Medicine, Affiliated Hospital of Jiangsu University, Zhenjiang, China; ^2^ Department of Pathogenic Biology, School of Medicine, Jiangsu University, Zhenjiang, China; ^3^ Department of Pathology and Center of Infection and Immunology, The University of Hong Kong, Hong Kong, China

**Keywords:** rheumatoid arthritis, microRNA, array, cytokine, chemokine, Immunology and Microbiology Section, Immune response, Immunity

## Abstract

The outstanding characteristics of circulatory microRNAs (miRNAs) attract much attention in research on disease biomarkers and disease pathogenesis. This study aimed to identify the expression profiles of plasma miRNAs in patients with rheumatoid arthritis (RA). Thirty-three miRNAs were screened using an miRNA array, of which 9 miRNAs were validated as differentially expressed in the plasma of RA patients compared with healthy controls (HCs). miRNA-4634 (miR-4634), miR-181d and miR-4764-5p expression levels were increased, whereas miR-342-3p, miR-3926, miR-3925-3p, miR-122-3p, miR-9-5p and miR-219-2-3p expression levels were decreased in RA patients. The areas under the curve (AUCs) were generated to estimate the sensitivity and specificity of each miRNA or the panel of all 9 miRNAs as biomarkers for RA. AUCs for 9 individual miRNAs ranged from 0.6254 to 0.818; however, the AUC for the panel of 9 miRNAs reached 0.964. Levels of miR-122-3p, miR-3925-3p, miR-342-3p and miR-4764-5p expression showed significant differences between RA and other control groups. miR-4764-5p, miR-4634, miR-9-5p and miR-219-2-3p exhibited significant correlations with either plasma cytokine and chemokine levels or clinical features. In conclusion, this study identified 9-plasma miRNAs signature in Chinese patients with RA which may serve as noninvasive biomarkers for the diagnosis of RA.

## INTRODUCTION

MicroRNAs (miRNAs) are small non-coding RNAs that have critical functions in a wide range of biological and pathologic processes [1, 2]. Recent studies demonstrated that miRNAs are exported from cells and are detected in the circulation including in serum, plasma, urine, saliva and other body fluids [3, 4]. Surprisingly, these circulatory miRNAs are well-protected from endogenous RNase activity and show a remarkable stability even under harsh conditions, including boiling, low/high pH, extended storage, and freeze-thaw cycles. Moreover, alterations in the expression of circulatory miRNAs have been associated with several diseases, such as cancers [5-8], autoimmune diseases [9-11] and tissue injury [12]. These outstanding characteristics of circulatory miRNAs have attracted much attention in disease biomarker and pathogenesis research.

Rheumatoid arthritis (RA) is a chronic inflammatory autoimmune disease of the synovial joints that is characterized by leukocyte infiltration and synoviocyte activation, which lead to cartilage and bone destruction [13, 14]. Recent studies have supported an important role for miRNAs in RA: aberrant miRNA expression has been detected in different cell types in RA, and these miRNAs regulate specific pathways involved in RA pathogenesis [15-19]. In contrast to cellular or tissue miRNAs, the expression profile of circulatory miRNAs in RA has not been fully investigated. Koichi Murata et al. selected 5 miRNAs and reported that plasma miR-132 differentiated healthy controls (HCs) clearly from patients with RA or osteoarthritis (OA), whereas synovial fluid miRNAs differentiated RA from OA [20]. In their subsequent report, using a TaqMan miRNA array containing 768 miRNAs, plasma miR-24 and miR-125a-5p were identified as potential biomarkers for RA [21].

In recent years, a growing body of new miRNAs has been identified, which provides new candidates for the screening of biomarkers of autoimmune diseases, including RA. In this study, we used the miRCURY™ LNA Array (v.18.0), a 7^th^-generation miRNA array containing 3100 capture probes, to evaluate the expression profiles of plasma miRNAs in Chinese RA patients and HCs. Nine plasma miRNAs were found to be differentially expressed which may serve as noninvasive biomarkers for the diagnosis of RA and their correlations with plasma cytokine, chemokine and clinical features may contribute to the further understanding of RA pathogenesis.

## RESULTS

### Study population

During the screening phase, 5 RA patients and 5 HCs were enrolled, and the characteristics of these study participants are presented in Table 1. The clinical features of the RA patients (*n* = 75) and HCs (*n* = 70) enrolled in the selection and validation sets are presented in Table 2. There was no significant difference in the distribution of age or gender between the RA and HC groups enrolled for screening, selection or validation.

**Table 1:** Clinical features of the RA patients and the HCs enrolled for the miRCURY™ LNA Array study

Characteristics	RA	HCs
Number of participants	5	5
Gender, male/female	0/5	0/5
Age, mean (range)	45.4(33 to 64)	43.8(30 to 65)
RF titer (IU)	202.56	NA
ESR (mm), mean (range)	25(12 to 37)	NA
CRP (mg/dl), mean (range)	12.36(1.46 to 26.3)	NA
DAS28, mean (range)	5.16(4.16 to 6.82)	NA
SJC, mean (range)	19(10 to 28)	NA
TJC, mean (range)	13(0 to 22)	NA

CRP, C-reactive protein; DAS28, 28-joint Disease Activity Score; ESR, erythrocyte sedimentation ratio; NA, not applicable; SJC, swollen joint count; TJC, tender joint count.

**Table 2:** Clinical features of the RA patients and HCs enrolled in the selection and validation sets

Characteristics	RA(active)	RA(non-active)	HCs
Number of participants	44	31	70
Gender, male/female	6/38	5/26	6/64
Age, mean (range)	53.3(24 to 75)	51.54(22 to 81)	46.67(26 to 68)
RF titer (IU)	332.7(8 to 1450	237.2(4 to 2560)	NA
ESR (mm), mean (range)	49.24(2 to 319)	28.48(4 to 250)	NA
CCP-Ab, mean (range)	275.28(20 to 942.7)	335.44(8 to 768)	NA
CRP (mg/dl), mean (range)	13.6(1 to 55.3))	9.2(1 to 64.7)	NA
DAS28, mean (range)	5.8(3.51 to 7.58)	2.1(1.18 to 3.18)	NA
SJC, mean (range)	7.8(2 to 20)	0.038(0 to 1)	NA
TJC, mean (range)	13.1(2 to 28)	0.21(0 to 2)	NA

RA, rheumatoid arthritis; HCs, healthy controls; CRP, C-reactive protein; DAS28, 28-joint Disease Activity Score; ESR, erythrocyte sedimentation ratio; NA, not applicable; SJC, swollen joint count; TJC, tender joint count.

### miRNA profiling in RA patients *vs.* HCs using the miRCURY™ LNA array

The miRCURY™ LNA Array (v.18.0), a 7^th^-generation miRNA array containing 3100 capture probes, was used to evaluate plasma miRNA expression profiles in RA patients and HCs. Each plasma sample from the enrolled RA patients and HCs (*n* = 5 each) was tested individually rather than pooling the samples, as is typically done. A total of 138 miRNAs (fold change ≥1.5) were up-regulated in RA, whereas 187 (fold change ≥1.5) were down-regulated. To identify differentially expressed miRNAs that were statistically significantly different, we performed Volcano-Plot filtering using data from the RA patients and HCs. The threshold used to screen up- or down-regulated miRNAs was a fold change ≥1.5 together with a *P*-value ≤0.05. Based on these criteria, 22 miRNAs were up-regulated and 11 miRNAs were down-regulated in RA plasma (see S1 Table). These 33 differentially expressed miRNAs between RA and HCs are shown as a heat map and using hierarchical clustering based on the Cq value of each miRNA (see S1 Figure).

### Validation of the array result by RT-PCR

To validate the array result, the 33 differentially expressed miRNAs screened by miRNA array were first selected in a small set of individual plasma samples (14 RAs and 18 HCs, selection phase) by qRT-PCR. miRNAs with a Cq value > 35 were excluded, and only miRNAs with a ≥1.5-fold mean change together with a *P*-value ≤0.05 were selected for further validation. Using these criteria, 22 differentially expressed miRNAs were selected (data not shown). Subsequently, the 22 selected miRNAs were further quantified in a larger cohort consisting of an independent set of individual plasma samples (75 RA and 70 HCs, validation set). As shown in Table 3, the levels of three miRNAs, miR-4634, miR-181d and miR-4764-5p, were significantly increased, whereas the levels of six miRNAs, miR-342-3p, miR-3926, miR-3925-3p, miR-122-3p, miR-9-5p, miR-219-2-3p, were significantly decreased in RA patients compared with HCs. For both the selection and validation phases, spike-in cel-miR-39 and cel-miR-238 were used for normalization.

**Table 3:** Differentially expressed miRNAs in the validation set determined using qRT-PCR (containing samples used in the selection phase)

	Gene symbol	Rank	Parametric P-value	HCs (n = 70)	RA (n = 75)	Fold-change
Up-regulated	hsa-miR-4634	1	0.0118	10.01	4.358	2.297
	hsa-miR-181d	2	0.0201	4.060	1.686	2.408
	hsa-miR-4764-5 p	3	0.0299	15.12	29.69	1.964
Down-regulated	hsa-miR-342-3p	1	0.0001	2.310	0.5078	0.2198
	hsa-miR-3926	2	0.0001	6.891	1.182	0.1715
	hsa-miR-3925-3p	3	0.0001	0.8896	0.04146	0.04661
	hsa-miR-122-3p	4	0.0207	13.27	2.729	0.2057
	hsa-miR-9-5p	5	0.0223	7.625	21.32	0.2796
	hsa-miR-219-2-3p	6	0.0331	16.18	3.768	0.2329

Ranked by P-values generated from statistical comparisons between RA patients and HCs. In the RA and HCs columns, values for the mean of 2^−Δcq^ for each group are listed. Fold-change is the ratio of relative expression of RA vs. HCs.

### Evaluation of the validated 9 miRNAs as biomarkers for the diagnosis of RA

To evaluate the potential application of the 9 miRNAs screened as biomarkers for RA, we generated ROC curves to estimate the sensitivity and specificity of each miRNA or the panel of all 9 miRNAs. As shown in Figure 2, the AUCs for 9 miRNAs ranged from 0.6254 to 0.818. Logistic regression was also used to evaluate the diagnostic value of the combination of the 9 miRNAs as a panel. The ROC curve for the 9-miRNA panel revealed a high diagnostic accuracy (AUC, 0.964; 95% confidence interval [CI], 0.932 - 0.997; *P* < 0.0001), which was better than that of the individual miRNAs. These results suggest that the combination of the 9 miRNAs in a panel shows great potential as a biomarker for discriminating RA patients from HCs.

**Figure 1:**
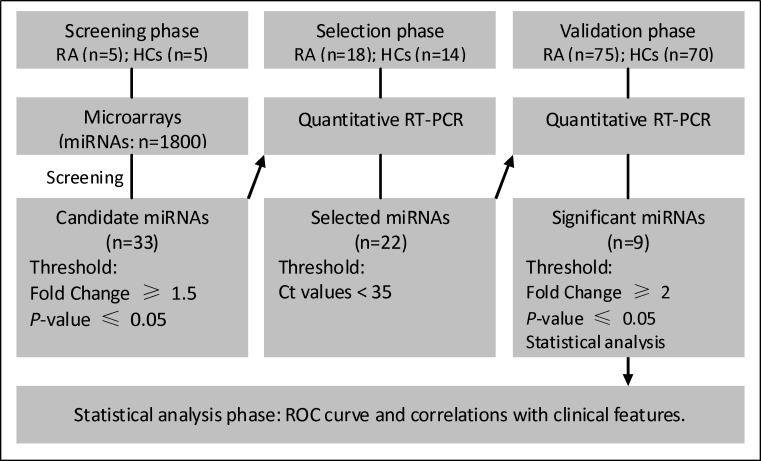
Overview of the experimental strategy RA, rheumatoid arthritis; HCs, healthy controls; RT-PCR, reverse transcriptase polymerase chain reaction; ROC, receiver operating characteristics.

**Figure 2:**
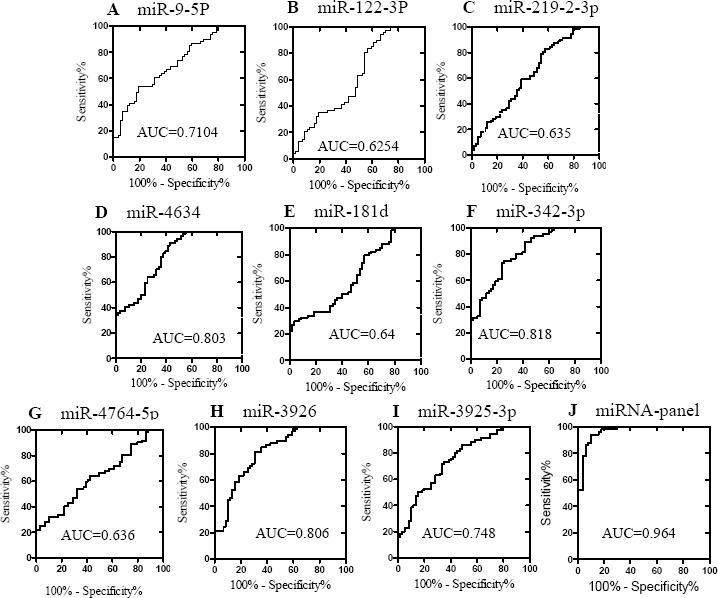
ROC curves showing the ability of the plasma concentrations of the 9 individual miRNAs (A-I) and the 9-miRNA panel (J) to differentiate the RA cases (*n* = 75) from the controls (*n* = 70)

### Evaluation of the validated 9 miRNAs for the discrimination of active RA from non-active RA

**Figure 3:**
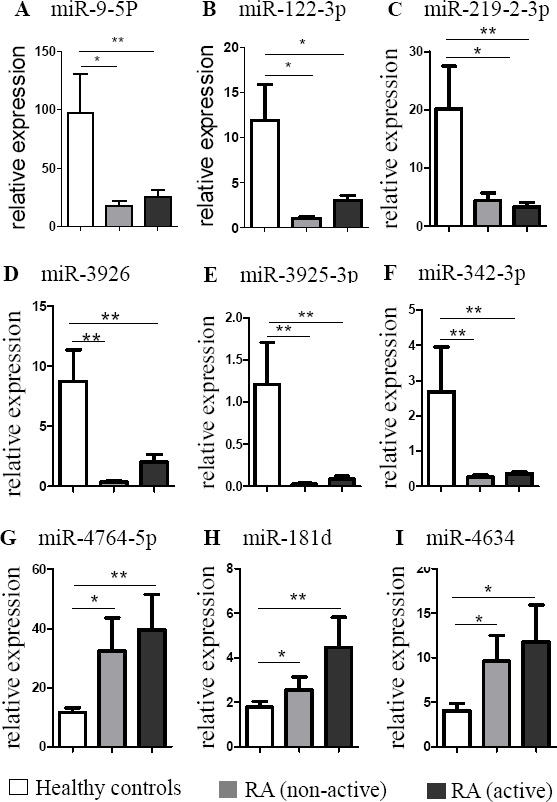
Evaluation of the validated 9 miRNAs for the discrimination of active RA from non-active RA RA patients were divided into two groups: the active group (*n* = 44) and the non-active group (*n* = 31) based on DAS28. The bars denote the mean 2^−Δcq^ values ± SD (*, *P*-values < 0.05 versus HCs; **, *P*-values < 0.01 versus HCs).

To further explore the potential usefulness of the 9 screened miRNAs as a biomarker, we analyzed the correlation of these plasma miRNAs with the clinical status of the RA patients. The RA patients were divided into an active RA group (*n* = 44) and a non-active group (*n* = 31) based on their DAS28 scores. Probability values were calculated using ANOVAs. Unexpectedly, active and non-active RA patients showed similar changes in expression of the 9 validated miRNAs compared with HCs. None of the 9 validated plasma miRNAs were differentially expressed between active and non-active RA (Figure 3). Our results suggest that these 9 validated plasma miRNAs are not suitable biomarkers for the discrimination of active vs. non-active RA.

### Evaluation of the validated 9 miRNAs for the discrimination of RA patients from disease controls

**Figure 4:**
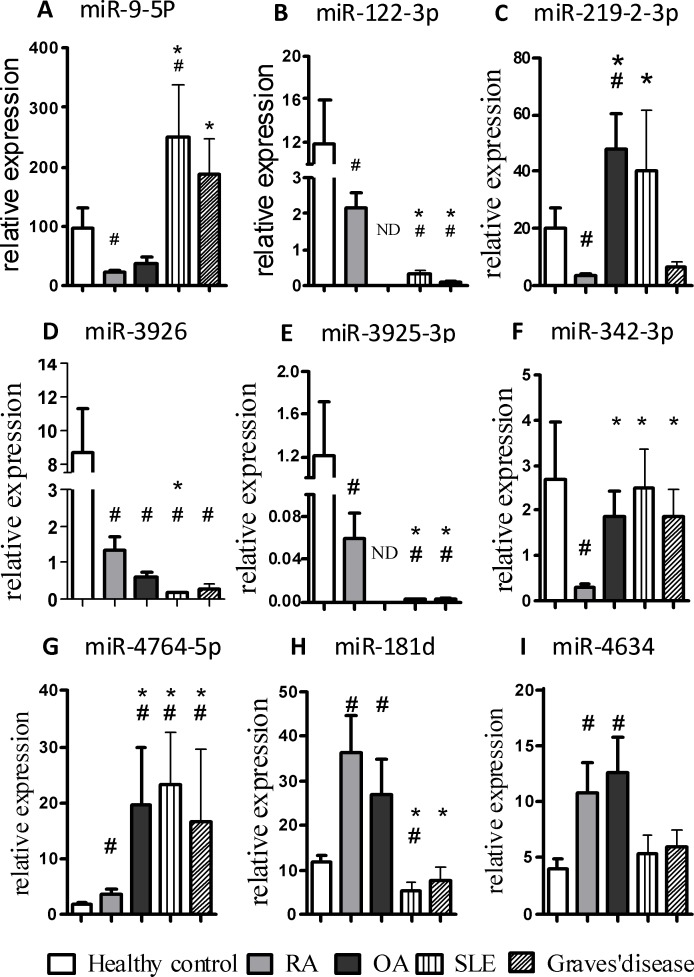
Evaluation of the validated 9 miRNAs for the discrimination of RA patients from disease controls The expression of 9 validated miRNAs in the plasma obtained from patients with RA (*n* = 75), OA (*n* = 18), SLE (*n* = 21), Graves' disease (*n* = 14) and HCs (*n* = 70) was determined by qRT-PCR. The bars denote the mean 2^−Δcq^ values ± SD (*, *P*-values < 0.05 versus RA; #, *P*-values < 0.05 versus HC. ND, None detected.)

The expression of 9 validated plasma miRNAs in patients with RA (*n* = 75, containing 44 active RA and 31 non-active RA patients) together with those from disease controls was also analyzed (Figure 4). 4 miRNAs showed highly significant differentiation between patients with RA and three groups of patients with other disease (OA, SLE, Graves' disease), in which the expression levels of miR-122-3p and miR-3925-3p were down-regulated while miR-342-3p and miR-4764-5p were up-regulated. In addition, miR-9-5p and miR-181d significantly discriminated the RA group from both SLE and Graves' disease groups but not the OA group. Moreover, miR-219-2-3p differentiated the RA group from the OA group but not SLE and Graves' disease group. Furthermore, miR-3926 could discriminate the RA group from the SLE group but not the OA and Graves' disease group. However, the expression levels of miR-4634 of RA patients did not show any significant differences from disease controls.

### Correlation between the 9 validated plasma miRNAs and the plasma cytokine and chemokine levels in RA

We next quantitatively measured the plasma cytokine and chemokine levels to assess their relationship to the 9 validated plasma miRNAs using Pearson's correlation analyses. The cytokines and chemokines measured were IL-6, IFN-γ, IL-1β, TNF-α, IL-17A, IL-4, IL-13, CXCL9, MCP-1, MIP-1α, MIP-1β and IL-8. miR-9-5p expression correlated positively with plasma levels of IFN-γ, TNF-α, IL-17A, IL-4 and CXCL9, whereas miR-219-2-3p expression only correlated significantly with CXCL9. miR-4764-5p expression was negatively correlated with IL-13. No significant correlations of the 6 other miRNAs with plasma cytokines or chemokines were noted in RA patients (see Table 4).

**Table 4:** Spearman rank correlations between the differentially expressed plasma miRNAs and cytokine and chemokine levels in RA patients (r/*P*)

Variable	miR-9-5p	miR-122-3p	miR-219-2-3P	miR-4634	miR-181d	miR-342-3p	miR-4764-5p	miR-3926	miR-3925-3p
**IL-6**	−0.2323 *p* = 0.2343	−0.2187 *p* = 0.2731	−0.06022 *p* = 0.7608	−0.03213 *p* = 0.8711	−0.07789 *p* = 0.6936	−0.09731 *p* = 0.6223	−02412 *p* = 0.2255	−02393 *p* = 0.2601	0.08707 *p* = 0.6723
**IL-1β**	0.2570 *p* = 0.1867	0.1688 *p* = 0.3906	−0.04032 *p* = 0.8386	−0.01826 *p* = 0.9280	0.09281 *p* = 0.6452	0.1979 *p* = 0.3127	−0.1579 *p* = 0.4222	0.06379 *p* = 0.7619	0.1256 *p* = 0.5408
**TNF-α**	0.4143 *p* = 0.0494	0.2376 *p* = 0.2528	−0.01801 *p* = 0.9319	0.08805 *p* = 0.6895	0.07644 *p* = 0.7165	0.3025 *p* = 0.1711	−0.1227 *p* = 0.5679	−0.1345 *p* = 0.5308	−0.2227 *p* = 0.2956
**IFN-γ**	0.4454 *p* = 0.0491	0.2380 *p* = 0.2861	−0.07470 *p* = 0.7411	0.07903 *p* = 0.7405	0.1049 *p* = 0.0506	0.2283 *p* = 0.3069	−0.1953 *p* = 0.3837	0.04103 *p* = 0.8561	0.1306 *p* = 0.5625
**IL-17A**	0.4349 *p* = 0.0431	0.3061 *p* = 0.1283	0.04851 *p* = 0.8140	0.1224 *p* = 0.5514	0.05285 *p* = 0.7976	0.2599 *p* = 0.1998	0.02056 *p* = 0.9206	0.1307 *p* = 0.5245	0.1656 *p* = 0.4189
**IL-4**	0.4158 *p* = 0.0278	0.1952 *p* = 0.2926	0.00465 *p* = 0.9802	0.0184 *p* = 0.9218	0.05747 *p* = 0.7588	0.1995 *p* = 0.2821	−0.1361 *p* = 0.4653	0.09976 *p* = 0.5934	0.2358 *p* = 0.2015
**IL-13**	0.02635 *p* = 0.9027	0.09804 *p* = 0.6486	−0.2185 *p* = 0.3051	−0.2185 *p* = 0.3049	−0.036635 *p* = 0.8651	0.1089 *p* = 0.6124	−0.4241 *p* = 0.0389	−0.1146 *p* = 0.594	0.0765 *p* = 0.7224
**CXCL9**	0.4737 *p* = 0.0009	0.2515 *p* = 0.0813	0.4055 *p* = 0.0085	0.2170 *p* = 0.1341	−0.03866 *p* = 0.7920	0.03672 *p* = 0.8022	0.1308 *p* = 0.3702	−0.1391 *p* = 0.3404	−0.1939 *p* = 0.1818
**MCP-1**	0.1403 *p* = 0.3312	0.04001 *p* = 0.7827	0.07996 *p* = 0.5810	−0.02246 *p* = 0.8770	0.01528 *p* = 0.9161	0.001168 *p* = 0.9936	0.07062 *p* = 0.6262	−0.1317 *p* = 0.3619	−0.1176 *p* = 0.4160
**MIP-1α**	0.1112 *p* = 0.4618	0.1054 *p* = 0.4859	0.05332 *p* = 0.7249	0.06869 *p* = 0.6501	0.01445 *p* = 0.9241	0.08785 *p* = 0.5616	0.2341 *p* = 0.1173	0.06175 *p* = 0.6835	−0.04892 *p* = 0.7468
**MIP-1β**	−0.03737 *p* = 0.8009	−0.07583 *p* = 0.6085	0.09176 *p* = 0.5351	0.04074 *p* = 0.7834	0.005932 *p* = 0.9681	0.03437 *p* = 0.8166	0.1698 *p* = 0.2486	0.01821 *p* = 0.9022	−0.02827 *p* = 0.08487
**IL-8**	−0.04628 *p* = 0.7949	−0.05651 *p* = 0.7509	0.03439 *p* = 0.8469	0.1248 *p* = 0.4818	−0.02513 *p* = 0.8878	0.01337 *p* = 0.9402	0.2542 *p* = 0.1468	0.09280 *p* = 0.6017	0.01076 *p* = 0.9518

### Correlations between the 9 validated plasma miRNAs and clinical variables in RA

We also investigated the correlations of the 9 validated miRNAs with clinical variables, including erythrocyte sedimentation rate (ESR), C-reactive protein (CRP), rheumatoid factor (RF), swollen joint count (SJC), tender joint count (TJC) and 28-joint Disease Activity Score (DAS28). Plasma miR-4634 correlated with all clinical features we examined, with the exception of CRP. Both miR-4764-5p and miR-219-2-3p correlated with RF, SJC, TJC and DAS28, whereas miR-9-5p correlated only with RF. Plasma miR-122-3p, miR-181d, miR-342-3p, miR-3926 and miR-3925-3p did not correlate with clinical parameters (see Table 5).

**Table 5:** Spearman rank correlations between differentially expressed plasma miRNAs and clinical features in RA patients (r/P)

Variable	miR-9-5p	miR-122-3p	miR-219-2-3P	miR-4634	miR-181d	miR-342-3p	miR-4764-5p	miR-3926	miR-3925-3p
**ESR**	−0.01187 *p* = 0.9355	0.03362 *p* = 0.8186	0.1900 *p* = 0.1910	0.4014 *p* = 0.0069	0.01802 *p* = 0.9022	−0.0084 *p* = 0.9545	0.07344 *p* = 0.6160	−0.1236 *p* = 0.3973	−0.1762 *p* = 0.2257
**CRP**	0.05934 *p* = 0.6855	0.09276 *p* = 0.5261	0.2202 *p* = 0.1284	0.1985 *p* = 0.1715	0.02157 *p* = 0.8830	0.05337 *p* = 0.7157	0.09069 *p* = 0.5354	−0.08076 *p* = 0.5812	−0.03299 *p* = 0.8220
**RF**	0.4635 *p* = 0.005	0.1408 *p* = 0.4059	0.412 *p* = 0.0172	0.4342 *p* = 0.0147	0.04564 *p* = 0.7885	0.1034 *p* = 0.5427	0.4699 *p* = 0.005	0.02072 *p* = 0.9031	−0.02438 *p* = 0.8861
**SJC**	−0.06894 *p* = 0.6379	−0.1299 *P* = 0.3736	0.4158 *p* = 0.0068	0.5010 *p* = 0.0004	0.1824 *p* = 0.2098	0.04737 *p* = 0.7466	0.4239 *p* = 0.0033	−0.1993 *p* = 0.1698	−0.1286 *p* = 0.3784
**TJC**	−0.02733 *p* = 0.7371	−0.1061 *p* = 0.4682	0.4037 *p* = 0.0066	0.4282 *p* = 0.0027	0.1473 *p* = 0.3124	0.1193 *p* = 0.4141	0.4015 *p* = 0.0047	−0.1779 *p* = 0.2215	−0.1274 *p* = 0.8330
**DAS28**	−0.01414 *p* = 0.9232	−0.06479 *p* = 0.6583	0.4358 *p* = 0.0068	0.5147 *p* = 0.0003	0.1224 *p* = 0.4023	0.01561 *p* = 0.9152	0.4228 *p* = 0.0028	−0.2504 *p* = 0.0827	−0.2238 *p* = 0.1222

## DISCUSSION

An increasing number of studies have addressed the important role of miRNAs in various physiological processes and the potential of miRNAs for use in clinical biochemistry. Although the utility of circulatory miRNAs as biomarkers remains exploratory, an effective diagnostic strategy has been developed. First, large-scale miRNA profiling (e.g., miRNA microarray, Solexa sequencing and miRNA TaqMan low-density arrays) is performed, and data are compared between a small group of patients with the disease of interest and a control group. miRNAs that show significant differences between groups are then selected and validated on larger populations using individual qRT-PCRs, thereby establishing performance parameters, such as sensitivity, specificity and AUCs. Thus far, following this strategy, previous miRNA array studies have identified many differentially expressed circulatory miRNAs as potential biomarkers in various diseases including cancers, autoimmune diseases and tissue injury [22-28].

In this current study, using an updated miRNA array containing 1800 miRNAs that was followed by qRT-PCR validation, we observed higher plasma expression of miR-4634, miR-181d and miR-4764-5p and lower expression of miR-342-3p, miR-3926, miR-3925-3p, miR-122-3p, miR-9-5p and miR-219-2-3p in Chinese RA patients. A previous study by Koichi Murata et al. also performed an array-based miRNA analysis of plasma samples from Japanese RA patients and HCs and identified increased concentrations of plasma miR-24 and miR-125a-5p, demonstrating their potential as diagnostic markers of RA [21]. There is no overlap in miRNA profiles between these two studies. Several reasons may underlie this unexpected result: first, the RA populations enrolled in these two studies were selected from different countries; thus, different genetic backgrounds may contribute partially to these differences. Secondly, Koichi Murata et al. assessed differential expression of plasma miRNAs in RA patients using an miRNA array containing 768 miRNAs, whereas in our study, we applied a larger array database containing 1800 miRNAs. In our study, several newly identified miRNAs were screened, although their functional characteristics remain unclear. In addition, for miRNA array analysis, we selected active RA patients who were not receiving clinical treatment rather than RA patients who were being treated, as were enrolled in the previous study by Murata et al [21].

The quantification of cellular or tissue miRNAs may be normalized to U6 small nuclear RNA (snRNA); however, thus far, there has been no suitable internal control for plasma miRNA normalization. The expression level of plasma miRNA is typically normalized by spiking in exogenous miRNA from *Caenorhabditis elegans* or plants as controls or is based on the volume of plasma sample. In this study, we spiked in two synthetic *Caenorhabditis elegans* miRNAs as exogenous controls (cel-miR-39 and cel-miR-238), which lacked sequence homology to human miRNAs, and we used the mean Cq value of these two external miRNAs to normalize plasma miRNA expression. This design may help reduce sample-to-sample variations. Although successful real-time RT-PCR technologies have been developed to amplify and quantify miRNAs, to apply plasma miRNAs as biomarkers in the clinical setting, it will be necessary to develop practical clinical assays that incorporate RNA extraction, primer design and normalization controls.

In this study, we identified a total of 9 miRNAs that were differentially expressed in the plasma of RA patients and HCs. Subsequently, we evaluated the potential of using these 9 miRNAs as diagnostic markers for RA. ROC curves were used to estimate the sensitivity and specificity of each miRNA. The AUCs for the 9 individual miRNAs ranged from 0.6254 to 0.818; however, the AUCs for the panel of the 9 miRNAs reached 0.964, which is significantly higher than those of the individual miRNAs. This result demonstrates that the combination of multiple plasma miRNAs is more accurate than individual miRNAs for RA diagnosis. Early studies evaluating miRNAs for use as biomarkers typically focused on individual miRNAs; however, the majority of recent studies tend to test the diagnostic value of miRNA panels in various human diseases. During the three stages of RA, which include autoimmune priming, tissue attack and chronic inflammation, there are many diverse, complex molecular events that may involve multiple miRNAs. Thus, multiple miRNAs, including joint tissue-specific miRNAs or miRNAs secreted by circulating blood cells, will be released into the circulation to form a specific plasma miRNA profile. Accordingly, these miRNAs may be screened and can serve as a biomarker panel for RA diagnosis. In this study, we compared the RA patients not only with HCs, but also with other disease controls, including OA, SLE and Graves' disease. Together, levels of 4 miRNAs, including miR-122-3p, miR-3925-3p, miR-342-3p and miR-4764-5p, were found to be statistically significant when compared with HCs and disease controls. Of note, the expression of miR-342-3p was specifically decreased in RA patients, indicating that miR-342-3p may serve as the best candidate biomarker for the diagnosis of RA.

The 9 plasma miRNAs identified in this study may be classified into two groups: an unknown function group (miR-4634, miR-4764-5p, miR-3926, miR-3925-3p) and a known function group (miR-181d, miR-342-3p, miR-122-3p, miR-9-5p, miR-219-2-3p). Although functional information on miRNAs in the former group is unavailable, our study demonstrates that miR-4764-5p plasma levels correlate positively with RF, SJC, TJC and DAS28 but negatively with IL-13. In addition, miR-4634 plasma levels correlated with all clinical features we examined with the exception of CRP. Further studies are required to identify the origin of these 4 plasma miRNAs and their target genes and the mechanisms regulating the biogenesis of these miRNAs. Although the plasma samples used for the array screening were from primary active RA patients, none of the 9 screened plasma miRNAs discriminated active RA from non-active RA. This unexpected result requires further confirmation in a larger cohort. Of the latter group, only two miRNAs, miR-9-5p and miR-219-2-3p, correlated with both cytokines and chemokine as well as clinical parameters. Notably, miR-9-5p is mainly correlated with immune/inflammatory cytokines while miR-219-2-3p with clinical variables. Although miR-9-5p has been reported as oncogenic microRNA in non-small cell lung cancer, large B-cell lymphomas, osteosarcoma and breast cancer[29-32], little evidence shows inter-regulation between miR-9-5p and cytokines. Recent studies have shown that miR-219-2-3p is associated with schizophrenia, diabetic retinopathy, acute inflammation and gastric cancer[33-37]. Thus, further studies are required to elucidate the possible mechanisms underlying the down-regulation and function of these two miRNAs in the peripheral blood of RA patients.

In summary, we have identified 9 differentially expressed plasma miRNAs in patients with RA that may serve as candidate biomarkers for RA diagnosis. In addition, several identified plasma miRNAs were found to be associated with clinical parameters, which may be implicated in the pathogenesis of RA disease. However, these data should be verified in a larger population of RA patients, and more importantly, functional experiments are required to validate the functional roles of these plasma miRNAs in RA pathogenesis.

## MATERIALS AND METHODS

### Study design

Three phases were designed to identify plasma miRNA profiles to act as potential biomarkers for RA (summarized in Figure 1). Briefly, in the initial biomarker screening stage, 10 plasma samples from 5 RA patients and 5 HCs were tested individually using the miRCURY™ LNA Array (version 18.0, Exiqon, Vedbaek, Denmark), a 7^th^-generation miRNA array containing 3100 capture probes, to identify miRNAs that were differentially expressed. In the second phase, differentially expressed miRNAs identified using the miRNA array were further selected using RT-qPCR in a small set of individual plasma samples (18 RAs and 14 HCs, selection phase). The selected miRNAs were then validated in a large number of individual samples from 75 RA patients and 70 HCs using qRT-PCR. A disease control cohort included 18 patients with OA, 21 patients with systemic lupus erythematosus (SLE)), 14 patients with Graves' disease. All of the study protocols and consent forms were approved by the Affiliated Hospital of Jiangsu University. All participants provided written informed consent to participate in this study.

### Plasma preparation, RNA isolation

Whole blood (2 mL) was collected from each subject, and plasma was separated by centrifugation at 1600 *g* for 10 min at room temperature followed by centrifugation at 16,000 *g* for 10 min at 4°C to remove all cell debris. The plasma supernatant was collected and stored at −80°C until further analysis.

For the miRNA array test, total plasma RNA was harvested using TRIzol (Invitrogen, Carlsbad, CA, USA)) and a miRNeasy Mini Kit (Qiagen, Valencia, CA, USA) according to manufacturer's instructions. For the RT-qPCR assay, total RNA was extracted from 200-μl volumes of plasma using the mirVana™ PARIS™ Kit (Ambion, Foster City, CA, USA) according to the manufacturer's instructions. Two synthetic *Caenorhabditis elegans* miRNAs, cel-miR-39 and cel-miR-238 (synthesized by Ribobio, Guangzhou, China), which lacked sequence homology to human miRNAs, were added to each denatured sample (after plasma was mixed with denaturing buffer) to normalize the sample-to-sample variation.

### miRCURY™ LNA array

The plasma miRNA expression profiles of 5 RA patients and 5 HCs were determined using the miRCURY™ LNA Array. RNA quantity was tested using a NanoDrop 1000, and the samples were then labeled using the miRCURY™ Hy3™/Hy5™ Power labeling kit and hybridized onto the miRCURY™ LNA Array. Following washing steps, the slides were scanned using an Axon GenePix 4000B microarray scanner. Scanned images were then imported into GenePix Pro 6.0 software (Axon Instruments, Foster City, CA) for grid alignment and data extraction. Replicated miRNAs were averaged, and miRNAs with intensities ≥30 in all samples were chosen for calculating the normalization factor. Data on miRNA expression were normalized using the median normalization protocol. Following normalization, significantly differentially expressed miRNAs were identified via Volcano Plot filtering. Finally, hierarchical clustering was performed using MEV software (v4.6, TIGR) to show distinguishing miRNAs among the samples.

### Determination of plasma miRNA by qRT-PCR

Two micrograms of total RNA were reverse-transcribed using a commercial kit (Promega, Madison, WI, USA) according to the manufacturer's protocol. Briefly, the 50-μl reactions were incubated for 60 min at 42°C and 10 min at 70°C and were then stored at 4°C. Real-time PCR analysis was performed using an Applied BioSystems 7500 Thermocycler according to manufacturer's protocol with SYBR Green PCR Master Mix (TaKaRa, Daliang, China). In short, the reactions were incubated at 95°C for 20 s, followed by 40 cycles of 95°C for 10 s and 60°C for 34 s. A dissociation stage was performed at the end of the amplification procedure to determine potential non-specific amplifications. Reverse transcription and qPCR were performed using a Bulge-Loop™ miRNA qPCR Primer Set (RiboBio, Guangzhou, China). The relative expression level of each miRNA was calculated using the comparative Cq (quantification cycle) method. To avoid possible differences due to amount of starting RNA, the resultant miRNA levels were normalized to the mean of cel-miR-39 and cel-miR-238. Data were analyzed using SDS Relative Quantification Software version 2.06 (Applied BioSystems, CA, USA).

### Cytokine and chemokine assays

The Human Th1/Th2/Th9/Th17/Th22 13plex FlowCytomix^TM^ kit and the Human Chemokine 6plex FlowCytomix kit from eBioscience (San Diego, CA, USA) were used to quantitatively measure levels of interleukin (IL)-1β, IL-2, IL-4, IL-6, IL-17A, interferon (IFN)-γ, tumor necrosis factor (TNF)-α, macrophage inflammatory protein (MIP)-1β, monocyte chemoattractant protein (MCP)-1, MIP-1α, IL-8 and chemokine (C-X-C motif) ligand 9 (CXCL9) in plasma according to the manufacturer's instructions.

### Data analysis and statistics

All expression values of plasma miRNAs were normalized to cel-miR-39 and cel-miR-238 and were then expressed as 2^−ΔCq^, in which ΔCq_miR_ = Cq_miR_-(Cq_cel-miR-39_+Cq_cel-miR-238_)/2. The miRNA data are presented as the means ± standard errors (SEs). For continuous data, comparisons between groups were performed using Student's t tests (two groups) or analyses of variance (ANOVAs) (>2 groups) for Gaussian data and using Mann-Whitney U tests (two groups) or Kruskal-Wallis tests (> 2 groups) for non-normally distributed data. Pearson's correlation analysis was used to assess relationships between plasma miRNA expression and chemokine and cytokine levels and the patients' clinical characteristics. Receiver-operator characteristic (ROC) curves and area under the curve (AUC) analyses were used to determine the sensitivity, specificity and corresponding cut-off values for each miRNA. Logistic regression was used to develop composite panels of biomarkers to identify a panel that could distinguish RA from control with the greatest sensitivity and specificity. A *p*-value was considered significant when less than 0.05. Statistical analyses were performed using SPSS software version 16.0.

## SUPPLEMENTARY MATERIAL TABLE AND FIGURE


