# Supplementary material for: Plasma microRNA expression profiles in Chinese patients with rheumatoid arthritis
Source: Oncotarget. 2015 Dec 2;6(40):42557–68. doi: 10.18632/oncotarget.6449 (PMC4767452; doi:10.18632/oncotarget.6449)
Supplement: Supplementary file 1 [file oncotarget-06-42557-s001.pdf]

Plasma microRNA expression profiles in Chinese patients with rheumatoid arthritis

Supplementary Material

Supplemental Table 1 Up- and down-regulated miRNAs in plasma samples from RA patients compared with HCs determined using the miRCURY™ LNA Array.

ID	Gene symbol	Rank	RAs	Controls	Fold-change	P-value
**Up-regulated miRNAs (n=22)**						
168878	hsa-miR-5100	1	1.000133	0.294362	3.397632	0.000613
169118	hsa-miR-5009-3p	2	0.11483	0.063456	1.809601	0.000645
168852	hsa-miR-4764-5p	3	3.426527	1.827897	1.874573	0.004911
168838	hsa-miR-720	4	0.419313	0.154747	2.709663	0.007281
169335	hsa-miR-4699-5p	5	0.114357	0.044225	2.585782	0.009556
42551	hsa-miR-122-3p	6	0.287497	0.129671	2.217132	0.010299
169310	hsa-miR-3913-3p	7	0.535137	0.295698	1.809741	0.010459
46380	hsa-miR-1255a	8	0.996117	0.612083	1.627423	0.010577
168849	hsa-miR-4635	9	0.120057	0.042092	2.852235	0.010942
148064	hsa-miR-3926	10	0.114758	0.059833	1.917982	0.013399
168872	hsa-miR-24-1-5p	11	0.160399	0.054158	2.961697	0.013492
169319	hsa-miR-3136-3p	12	2.877488	1.54198	1.866099	0.01384
169140	hsa-miR-3150a-5p	13	5.961167	3.024471	1.970978	0.020444
168616	hsa-miR-4536-3p	14	0.090053	0.038444	2.342417	0.022176
169072	hsa-miR-3925-3p	15	0.19699	0.128511	1.532867	0.024553
168834	hsa-miR-5571-5p	16	0.368272	0.228677	1.610449	0.024682
168650	hsa-miR-3121-5p	17	0.265737	0.166803	1.593113	0.025936
168627	hsa-miR-4678	18	1.123368	0.286157	3.925703	0.035873
17529	hcmv-miR-US25-2-3p	19	0.169993	0.085289	1.993149	0.046009
169411	hsa-miR-205-3p	20	1.875961	1.106103	1.696009	0.04745
30033	hsa-miR-877-5p	21	2.275638	1.221841	1.862466	0.048146
145831	hsa-miR-625-5p	22	6.350872	1.544279	4.112517	0.04913
**Down-regulated miRNAs (n=11)**						
169408	hsa-miR-181d	1	0.259705	0.476157	0.545418	0.000661
42957	hsa-miR-323a-3p	2	0.093783	0.232713	0.403	0.007403
145637	hsa-miR-187-3p	3	0.751956	1.29768	0.579462	0.013015
32884	hsa-miR-342-3p	4	0.078643	0.150178	0.523665	0.014757
169263	hsa-miR-4634	5	0.013533	0.134088	0.100924	0.022005
4040	hsa-miR-9-5p	6	0.768069	2.06374	0.372173	0.039091
146159	hsv1-miR-H4-3p	7	3.930276	9.655809	0.407037	0.039308
42834	hsa-miR-219-2-3p	8	0.137126	0.425542	0.322238	0.041348
145943	hsa-miR-100-5p	9	0.13703	0.329753	0.415554	0.041422
10928	hsa-miR-125a-5p	10	1.627243	2.672568	0.608869	0.042549

169376	hsa-miR-5701	11	0.496877	0.048581	0.355479	0.715426

Data are expressed as the medians. ID: array ID of the probes; *P*-value, T-test results from comparisons between RA patients and HCs; Rank, Ranked by *P*-value in screening phase; Fold change, the ratio of normalized intensities between samples of the different groups.

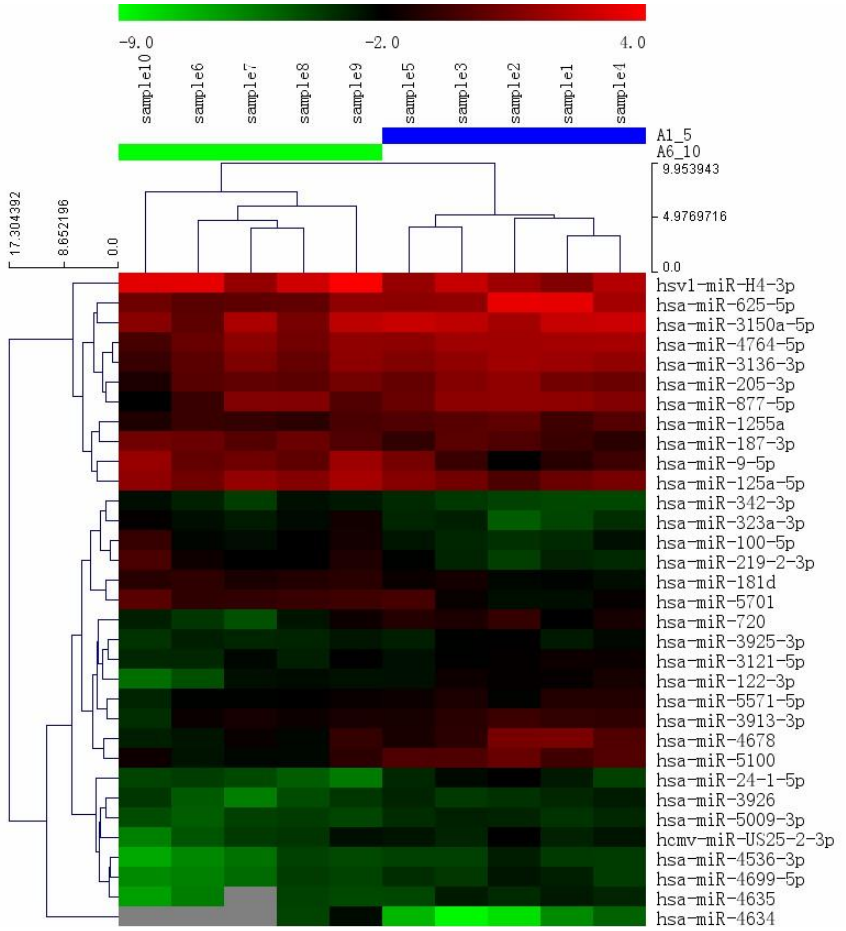

Supplemental Fig. 1 miRNA screening using the miRCURY™ LNA Array. Heat map clustering of the miRNA microarray data from the RA patients and controls; HCs (blue) and RA (green) showing the up-regulated miRNAs (in red; the majority of changes) and the down-regulated miRNAs (in green; fewer changes) in RA patient plasma. The heat map diagram shows results from two-way hierarchical clustering of miRNAs and samples. Each row denotes an individual miRNA, and each column denotes a sample. The miRNA clustering tree is shown on the left, and the sample clustering tree appears at the top. The color scale shown at the top illustrates the relative miRNA expression levels using grading: a red color denotes high relative expression levels, and a green color denotes low relative expression levels.
